# Altered Swimming Behaviors in Zebrafish Larvae Lacking Cannabinoid Receptor 2

**DOI:** 10.1089/can.2018.0025

**Published:** 2019-06-14

**Authors:** Agnes Acevedo-Canabal, Luis Colón-Cruz, Roberto Rodriguez-Morales, Gaurav K. Varshney, Shawn Burgess, Lorena González-Sepúlveda, Guillermo Yudowski, Martine Behra

**Affiliations:** ^1^ Department of Anatomy and Neurobiology, Medical Sciences Campus, University of Puerto Rico (MSC-UPR), San Juan, Puerto Rico.; ^2^ Department of Anatomy and Neurobiology, Institute of Neurobiology, University of Puerto Rico, San Juan, Puerto Rico.; ^3^ Oklahoma Medical Research Foundation, Oklahoma City, Oklahoma.; ^4^ National Human Genome Research Institute, NHGRI/NIH, Bethesda, Maryland.; ^5^ Puerto Rico Clinical and Translational Research Consortium, Medical Sciences Campus, University of Puerto Rico (MSC-UPR), San Juan, Puerto Rico.

**Keywords:** cannabinoid receptor 2 knockout, PTZ, VPA, zebrafish larva, JWH-133, AM-360

## Abstract

**Background and Objectives:** The cannabinoid receptor 2 (CB2) was previously implicated in brain functions, including complex behaviors. Here, we assessed the role of CB2 in selected swimming behaviors in zebrafish larvae and developed an *in vivo* upscalable whole-organism approach for CB2 ligand screening.

**Experimental Approach:** Using CRISPR-Cas9 technology, we generated a novel null allele (*cnr2^upr1^*) and a stable homozygote-viable loss-of-function (CB2-KO) line. We measured in untreated wild-type and *cnr2^upr1/upr1^* larvae, photo-dependent (swimming) responses (PDR) and center occupancy (CO) to establish quantifiable anxiety-like parameters. Next, we measured PDR alteration and CO variation while exposing wild-type and mutant animals to an anxiolytic drug (valproic acid [VPA]) or to an anxiogenic drug (pentylenetetrazol [PTZ]). Finally, we treated wild-type and mutant larvae with two CB2-specific agonists (JWH-133 and HU-308) and two CB2-specific antagonists, inverse agonists (AM-630 and SR-144528).

**Results:** Untreated CB2-KO showed a different PDR than wild-type larvae as well as a decreased CO. VPA treatments diminished swimming activity in all animals but to a lesser extend in mutants. CO was strongly diminished and even more in mutants. PTZ-induced inverted PDR was significantly stronger in light and weaker in dark periods and the CO lower in PTZ-treated mutants. Finally, two of four tested CB2 ligands had a detectable activity in the assay.

**Conclusions:** We showed that larvae lacking CB2 behave differently in complex behaviors that can be assimilated to anxiety-like behaviors. Mutant larvae responded differently to VPA and PTZ treatments, providing *in vivo* evidence of CB2 modulating complex behaviors. We also established an upscalable combined genetic/behavioral approach in a whole organism that could be further developed for high-throughput drug discovery platforms.

## Introduction

The endocannabinoid (eCB) system is a key modulator of excitatory and inhibitory neuronal activity^1^ and its dysregulation has been linked to several psychiatric disorders.^2–5^ The two cannabinoid receptors (CB1 and CB2) belong to the G protein-coupled receptor family. They are both activated by endogenous ligands (eCBs)^6,7^ and exogenous compounds such as Δ^9^-THC, the main psychoactive component in *cannabis*.^8^ CB1 is highly expressed in the CNS and implicated in numerous neurological diseases (for review, see Marco et al.,^3^ Kendall and Yudowski,^5^ Bilkei-Gorzo,^9^ Di Marzo et al.,^10^ and Pavlopoulos et al.^11^). By comparison, CB2 expression was initially described in the immune system but more recently also in discreet brain regions where its role is still poorly understood.

A genome-wide association study showed association between specific SNPs in the *CNR2* gene encoding CB2 and schizophrenia.^12^ Several lines of evidence suggest a role for CB2 in complex and specific behaviors in adult rodents.^13–15^ CB2-KO mice displayed schizophrenia-related behaviors^16^ altered cognitive function,^17,18^ modified cocaine-reward behaviors,^19^ as well as increased aggressiveness.^20^ CB2 overexpression showed reduced anxiety-like behaviors^21^ and resistance to depression,^22^ whereas temporary blockage of CB2 expression exhibited reduced aversion to open space.^15^ Conditional CB2-KO demonstrated that CB2 can regulate synaptic transmission in hippocampal pyramidal cells and modulate gamma oscillation.^23^ Modulation of CB2 expression in the hippocampus showed a regulatory role in fear and working memory.^18^ Suppression of CB2 expression in dopamine neurons inhibited psychomotor behaviors, altered anxiety, and depression measurements, as well as alcohol preferences.^24^ So far, few developmental studies were performed for CB2^25,26^ and none was exploring complex behaviors during this critical period.

Zebrafish is a powerful, genetic, and developmental model, which also provides the unique feature of upscalability, allowing high-throughput applicable to pharmacological screens (for review, see Rennekamp and Peterson^27^). Using CRISPR-Cas9, we created a novel null allele (*cnr2^upr1^*) and established a stable loss-of-function (CB2-KO) zebrafish mutant line.^28^ Homozygote larvae were viable without an overt phenotype and were raised into fertile and healthy adults over several generations, which were all completely lacking CB2. Next, we tested swimming behaviors in 6-day postfertilization (dpf) wild-type and homozygote larvae monitoring photo-dependent responses (PDR)^29,30^ and measuring the center occupancy (CO) of wells providing an inverse measure of center avoidance. We found that CB2-KO were swimming significantly less in light and significantly more in dark periods with a decreased CO, when compared with wild-type larvae. When adding a broad-spectrum anxiolytic drug (valproic acid [VPA])^30,31^ just prior recording, we found that swimming activity and CO were strongly reduced in all animals in a similar manner, but swimming was slightly less and CO more diminished in mutants. When adding a classical anxiogenic drug, pentylenetetrazol (PTZ), a well-characterized GABA_A_ inhibitor in many animal models,^32–36^ we found that larvae lacking CB2 presented an increase in swimming activity and a decrease in CO when compared with wild type. Taken together, we provide *in vivo* evidence for CB2 modulating complex behaviors in zebrafish larvae.

Finally, to test the potential of our approach for CB2 ligand screening, we treated wild-type and CB2-KO larvae with two CB2-specific agonists (JWH-133 and HU-308) and two CB2-specific antagonists (AM-630 and SR-144528).^37^ We found that two of four treatments elicited detectable PDR alterations, possibly CB2 mediated for the most part. Thus, we described a novel, upscalable behavioral approach for drug screening in a whole organism, providing a complementary alternative to current methods.

## Materials and Methods

### Zebrafish care and husbandry

We used TAB5 or NHGRI wild-type animals that we raised and maintained in our fish room following standard procedures and IACUC protocol (#A880216).

### CRISPR-Cas9 generation of the *cnr2^upr1^* allele

We designed a CRISPR-Cas9 guide targeting 5′-ATGGCGTTTACGGGCTCTGT-3′ in *cnr2* (ENSDARG00000039970).^28,38^ Progeny were screened for insertion/deletion (INDEL) by polymerase chain reaction (PCR) amplification and sequencing with the following primers (F: 5′-GACCACACAAGAGCAGAAAGC-3′; R: 5′-GACGATCCAACCAGGTTTTG-3′), and by fluorescent-based PCR fragment analysis performed with fluorescent primers (F: 5′-CGCCCATCGTACCTGTTTAT-3′; R: 5′-TTGGCTCTAGTGCGTGTCAG-3′^39^).

### CB2 mRNA and protein expression

Total RNA was prepared from genotyped larvae and the subregion of interest in the *cnr2* mRNA transcribed using retrotranscriptase (Sigma) and the following primers (F: 5′-CAGCTGCCACGTGATATAAGTA-3′; R: 5′-ATGCCAGCATTTCTCCCCTC-3′), and subsequently sequenced with the same primers.

For Western blots, adult wild-type and *cnr2^upr1/upr1^* brains were dissected and digested in radioimmunoprecipitation assay buffer (Thermo Fisher Scientific #89900), supplemented with protease and phosphatase inhibitor cocktail (100×) (Thermo Fisher Scientific #78440). A dilution of 1:500 was used for the anti-CB2 antibody (Ab; Cayman Chemicals #101550) and 1:1000 for the anti-alpha-tubulin Ab (Developmental Studies Hybridoma Bank #12G10).

### The behavioral swimming assay: the PDR

Five dpf larvae were loaded into 48-well plates (CELLSTAR^®^) 24 h prior recording, for animals to adapt to the new environment. A single larva was placed in each well in 450 μL system water (SW). Next day, wells were topped off to 500 μL with SW and the desired concentration of the drug at study or with SW, and plates were immediately placed in the recording device (Zebrabox, Viewpoint, France). After a 30-min adaptation/incubation in the dark, animals were subjected to 10 min of light (L) at maximum intensity (=385 Lux) followed by 10 min of dark (D) in four successive cycles. All results were binned into 1-min intervals.

### Center occupancy

We virtually defined an inner central diameter (0.5 cm) within the whole well (1.0 cm) and recorded inner and total traveled distances. The percentage of CO was calculated as follows: CO=inner traveled distance/total traveled distance×100.

### Drug preparation

Stock solutions were prepared in SW at 20 mM for VPA (Sigma #P4543) and 75 mM for PTZ (Sigma #P6500),^30^ or in dimethyl sulfoxide at 10 mM for all CB2 ligands (Tocris bioscience: JWH-133 #1343, HU-308 #3088, AM-630 #1120, and SR-144528 #5039). Further dilutions were done in SW.

### Toxicity/survival assay

CB2 ligands Kis are in the nM range. However, to overcome diffusion problem and permeability issues, we expected to use them in the μM range. To exclude aberrant swimming patterns induced by overexposure compromising health and survival, we treated healthy 5 and 6 dpf larvae with CB2 ligands at 1, 10, and 50 μM (*n*=10/ligand/concentration) for 24 h and assessed the following criteria: overall larval morphology, spontaneous swimming, and responses to sound and mechanical stimuli. Concentrations resulting in abnormal morphology or responses were considered not well-tolerated and excluded from further experiments.

### Statistical analysis

We analyzed averaged total traveled distances per larva (with a minimum of triplicate experiments and 24 animals/treatment) in GraphPad Prism (v.7). All results were binned into 1-min intervals and error bars represent mean±standard error of the mean. Statistical differences between direct comparisons were calculated using multiple t-tests controlling the effect of the correlation among the number of fixed repeated measures. We performed two-way analysis of variance in graphs when two or more groups were compared simultaneously. Differences with *p*<0.05 were considered significant (*).

## Results

### Generation of a CB2 loss-of-function stable mutant line (null allele: *cnr2^upr1^)*

**FIG. 1.**
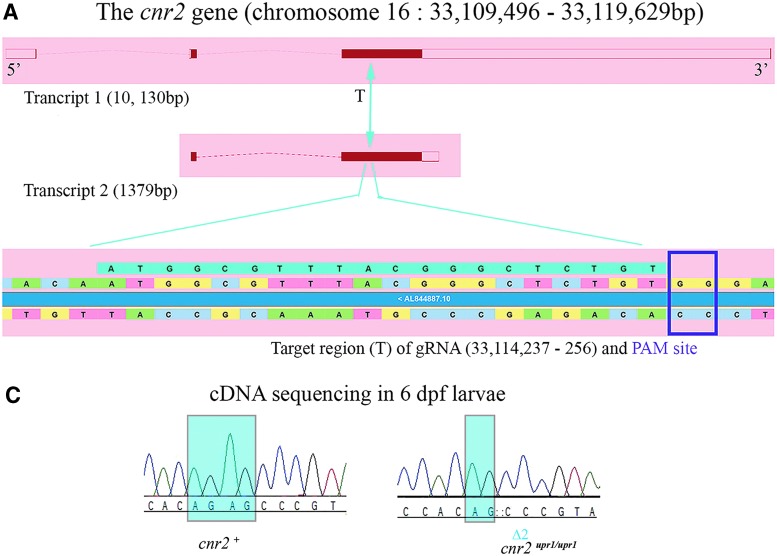

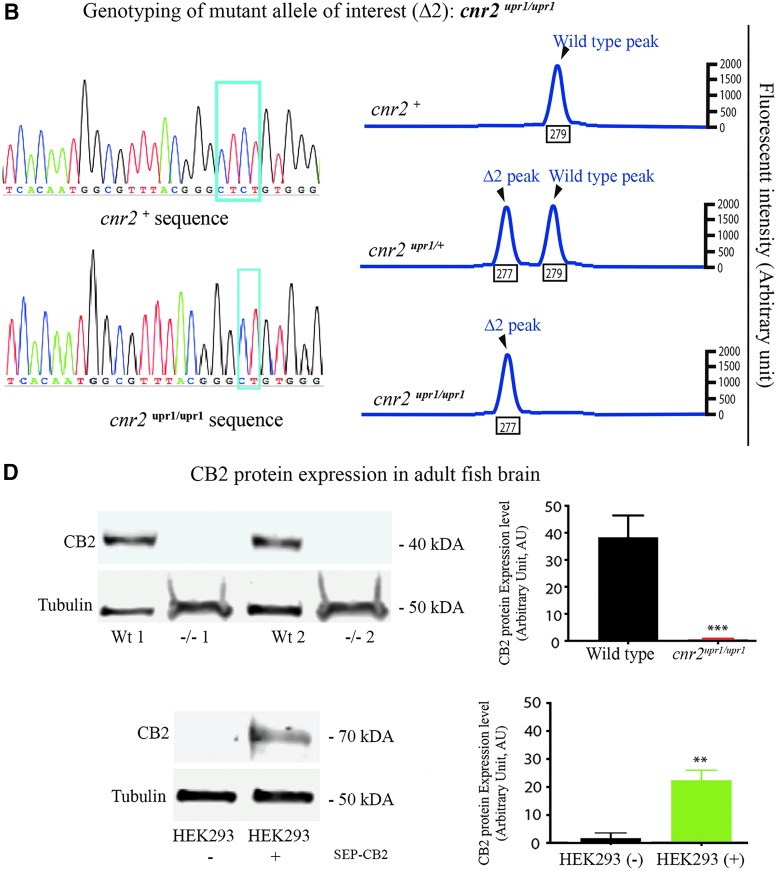
Generation of a CB2 loss-of-function stable mutant line (null allele: *cnr2^upr1^*). **(A)** Genomic environment of *cnr2* on zebrafish chromosome 16 showing two transcripts of 10,130 bp (top) and 1379 bp (middle). Translated exons are shown as solid red blocks. The CRISPR-Cas9 targeted region (T) is enlarged in lower panel (underlined in aqua blue) and the PAM site (purple square) is indicated. **(B)** Representative example of sequencing results (left panels) showing chromatographs of a wild-type larva (top, *cnr2^+^*) and a mutant larva (bottom, *cnr2^upr1/upr1^*) with the 2 bp deletion (Δ2: CT, blue square). Representative fluorescent polymerase chain reaction results (right panels) detecting fluorescence of amplicons according to their respective length. The wild-type (top) amplicon is 279 bp (single peak at 279). Heterozygotes (middle, *cnr2^upr1/+^*), carrying both the wild-type (279) and the mutant (Δ2, [279–2=277] alleles, show two separate peaks of equal intensity, 277 and at 279). Homozygotes (bottom, *cnr2^upr1/upr1^*) only carry the mutant allele (Δ2) with a single peak at 277. **(C)** Representative sequencing results of retrotranscribed cDNA from *cnr2* mRNA present in total RNA preparations from wild-type *(cnr2^+^,* left panel) and homozygote (*cnr2^upr1/upr1^*, right panel) genotyped larva show in the target region the expected Δ2: GA. **(D)** Western blots with anti-CB2 Ab against human CB2 cross react with zebrafish CB2. Protein extracts prepared from dissected wild-type (Wt1, Wt2) and genotyped mutant homozygote *cnr2^upr1/upr1^* (−/−1, −/−2) adult fish brains (upper left panels). The expected 40 kDA band was found in both the wild-type extracts, but absent in both mutant extracts. Validation of the anti-CB2-Ab was performed with protein extracts prepared from nontransfected HEK293 cells (HEK293-), and fluorescent CB2 expression construct (SEP-CB2)-transfected cells show the expected band at 70 kDA (left lower panels) in transfected cells only. Tubulin expression was probed as loading control (arbitrary unit, right panels). Error bars represent the standard errors of the mean (SEM). Statistical significance, **p<0.01 and ***p<0.001. Ab, antibody; bp, base pair.

In zebrafish, a single *cnr2* gene with two transcripts (Fig. 1A) resulting in identical translated exons (solid red blocks) encodes a 383 amino acid (aa) CB2 protein, which is slightly longer than the human homologue (360 aa). To generate loss-of-function alleles, we designed guide RNAs targeting the 5′ end of the second translated exon (Fig. 1A, T in light blue).

We outcrossed adult founders (F_0_) and genotyped the offspring (F_1_) for germ line transmission of INDEL in the target site. We identified a two-nucleotide deletion (Δ2: CT) just 3 base pair upstream of the protospacer adjacent motif site (purple square in bottom of Fig. 1A) introducing a translation frameshift, which would predictably create an early stop codon in aa position 159. The resulting truncated protein, if not degraded, would have only two transmembrane domains, thus very likely a loss-of-function mutation. We grew this allele (*cnr2^upr1^*) to homozygosity and genotyped animals by classical sequencing (Fig. 1B, left panels) or by fluorescent PCR^40^ (Fig. 1B, right panels). We closely monitored heterozygote or homozygote larvae development and morphology between 2- and 9-dpf and found no obvious phenotype. Likewise, adult genotyped *cnr2^upr1/upr1^* animals were healthy, fertile, and inbred into a stable F_3_ generation, from which we obtained *cnr2^upr1/upr1^* larvae used in all the behavioral studies.

To confirm that we had generated a loss of function, we analyzed the *cnr2* gene products. First, we synthetized and sequenced the cDNA obtained from 6 dpf wild-type (*n*=3) and genotyped *cnr2^upr1/upr1^* (*n*=3) single larvae, all of which carried the deletion (Δ2: CT, Fig. 1C). Next, we performed Western blots with an anti-CB2 Ab raised against an epitope located before the first transmembrane domain of human CB2, to allow detection of a putative truncated protein. We prepared protein extracts from dissected adult brains of wild-type (*n*=3), and genotyped *cnr2^upr1/upr1^*(*n*=3) animals. As predicted, we found a CB2-specific band at ∼40 kDa in all wild-type (Fig. 1D, top left panels, Wt1 and Wt2 are shown) but not in any *cnr2^upr1/upr1^* extracts (−/−1 and −/−2 are shown). Notably, no shorter CB2-KO-specific product was found, arguing that the truncated protein was unstable. We further validated specificity of the CB2-Ab in HEK293 cells (Fig. 1D lower left panels), which we transfected with a tagged CB2 construct (SEP-CB2) detected at 70 kDA. We probed tubulin expression in all samples (Fig. 1D, lower bands in left panels) and quantified it to determine the relative CB2 expression (graphs on the right). Taken together, we demonstrated that CB2 was expressed in adult fish brain but absent in *cnr2^upr1/upr1^*, and that it was a null allele resulting in viable homozygote larvae and adult totally devoid of CB2.

### Absence of CB2 affects the swimming PDR

**FIG. 2.**
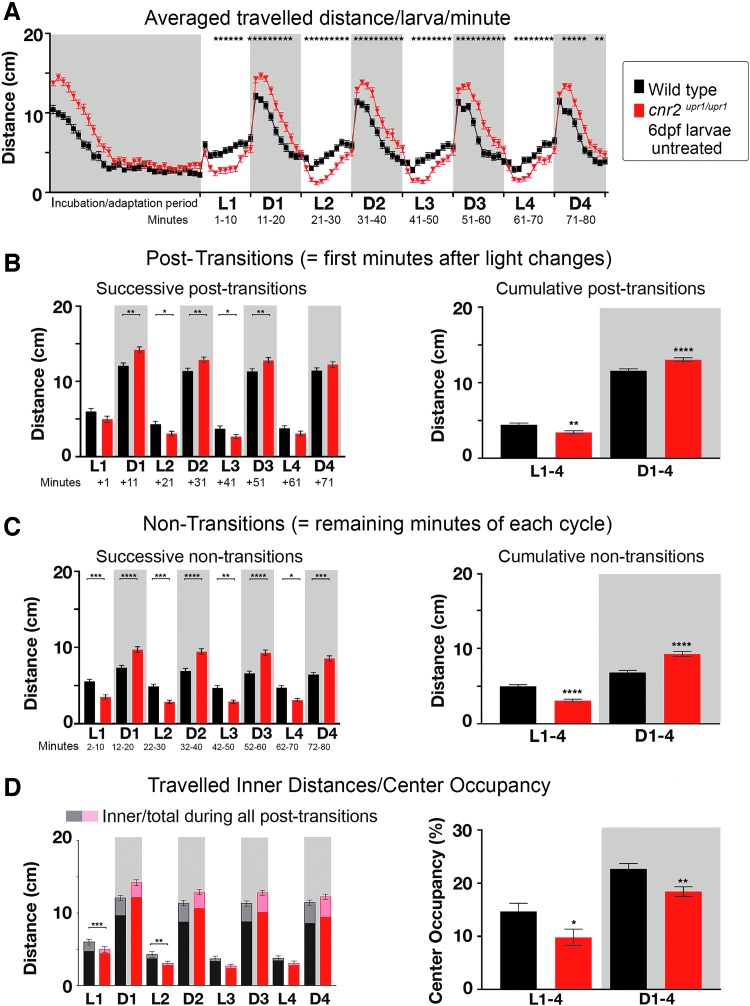
PDR in untreated 6 dpf wild-type and *cnr2^upr1/upr1^* larvae. **(A)** Averaged distance traveled per minute by 6 dpf wild-type (black line and squares) and *cnr2^upr1/upr1^* larvae (red line and triangles) submitted to four successive cycles of 10 min of alternating light periods (L1 to L4, white boxes) and dark periods (D1 to D4, gray boxes) after a 30-min incubation/adaptation period to dark. **(B)** Distances traveled in all successive (left panel) and cumulative (right panel) post-transitions by wild-type (black bars) and *cnr2^upr1/upr1^* (red bars) larvae. **(C)** Distances traveled during all successive (left panel) and cumulative (right) nontransitions. **(D)** Inner distances (in lighter color) traveled in successive nontransitions sur-imposed on total traveled distances (left panel). Percentage of CO calculated as follows: inner/total distance traveled×100. Error bars represent the standard errors of the mean (SEM). Statistical significance, *p<0.05, **p<0.01, ***p<0.001, and ****p<0.0001, and for clarity ns is omitted. CO, center occupancy; dpf, day postfertilization; ns, not significant; PDR, photo-dependent responses.

To determine if CB2 could have a role in complex behaviors, we assessed swimming behaviors using a previously established PDR assay,^29^ in which we measured traveled distance and CO during four successive 10-min-long light (L) and dark (D) periods. We recorded simultaneously wild-type and mutant larvae individually distributed in 48-well plates. L/D cycling was started after 30 min of adaptation to darkness. We graphed the averaged traveled distance/larva/min for the entire recording time (Fig. 2A, Wt black and KO red, *N*=5, *n*=120 larvae/genotype). A highly reproducible swimming pattern (=PDR) emerged: lower swimming activity in L, which strongly increased in D periods. Most drastic changes were always occurring immediately after a light change and leveling out over the remainder of the period. Thus, we decomposed the analysis into successive (left panels) and cumulative (right panels) post-transition (=first min after an L/D or D/L change, Fig. 2B), and nontransition (=remainder of a period, Fig. 2C).

**FIG. 3.**
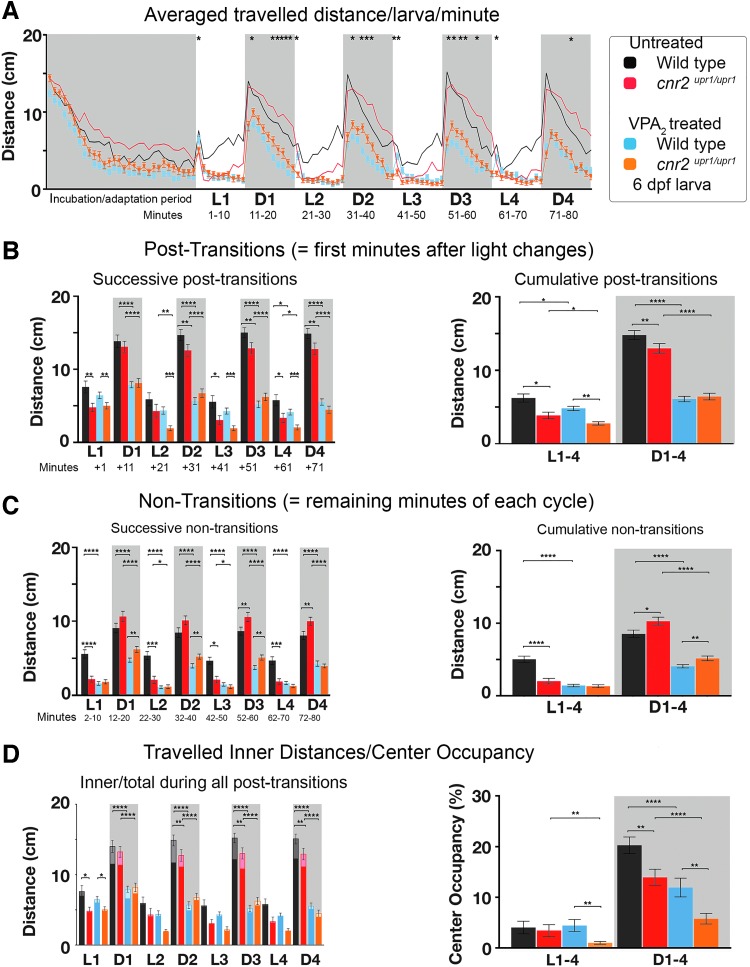
PDR in untreated and VPA [2 mM] (VPA_2_)-treated wild-type and *cnr2^upr1/upr1^* 6 dpf larvae. **(A)** Averaged distance traveled per minute by VPA_2_-treated wild-type (blue line and squares) and VPA_2_-treated *cnr2^upr1/upr1^* (orange line and triangles), and by untreated wild-type (black line) and *cnr2^upr1/upr1^* larvae (red line) represented in filigree. **(B)** Distances traveled in all successive (left panel) and cumulative (right panel) post-transitions by untreated wild-type (black bars) and *cnr2^upr1/upr1^* (red bars), and VPA_2_-treated wild-type (blue bars) and *cnr2^upr1/upr1^* (orange bars) larvae. **(C)** Distances traveled during all successive (left panel) and cumulative (right) nontransitions. **(D)** Inner distances (in lighter color) traveled in successive nontransitions sur-imposed on total traveled distances (left panel). Percentage of CO calculated as follows: inner/total distance traveled×100. Error bars represent the SEM. Statistical significance in **(A)** compares treated wild type versus treated mutant, in all other graphs as indicated by bracket. *p<0.05, **p<0.01, ***p<0.001, and ****p<0.0001, and for clarity ns is omitted. SEM, standard error of the mean; VPA, valproic acid.

Notably, *cnr2^upr1/upr1^* traveled significantly less in L and significantly more in D periods than wild-type animals (* in Fig. 2A), displaying a clear genetic-dependent PDR. In L periods, mutant larvae traveled slightly, but significantly less in cumulative post-transitions (right panel, white Wt=4.45 cm/min vs. KO=3.43 cm/min, *p*<0.001) as well as in all nontransitions (Fig. 2C, successive: left panel white, *p*<0.05; cumulative: right panel white Wt=4.97 cm/min vs. KO=3.08 cm/min, *p*<0.0001). In D periods, mutant traveled significantly more in post-transitions (Fig. 2B, successive: left panel gray, *p*<0.01 except in D4; cumulative: right panel gray Wt=6.80 cm/min vs. KO=9.25 cm/min, *p*<0.001) and in all nontransitions (Fig. 3C, successive: left panel gray, *p*<0.001; cumulative: right panel gray Wt=11.57 cm/min vs. KO=13.04 cm/min, *p*<0.0001). Thus, mutant animals were significantly less active in L and more active in D periods, suggesting a possible CB2 modulation of light-dependent hypo/hyperactivity.

Inner traveled distances were not significantly and consistently different between wild-type and mutant larvae (Fig. 2D left panel), but when we calculated the CO, it was significantly lower in mutants in D/L (right panel white CO_Wt_=14.70% vs. CO_KO_=9.80%, *p*<0.05) and L/D post-transitions (gray CO_Wt_=22.70% vs. CO_KO_=18.40%, *p*<0.001). Thus, CB2-KO animals spent less time in the center independently of the total distance traveled, suggesting that mutant larvae were avoiding open spaces more than wild-type animals. Taken together, animals lacking CB2 were hypoactive in L, hyperactive in D periods, and had decreased CO. Those results led us to postulate that CB2 was modulating complex behaviors.

### Animals lacking CB2 respond differently to anxiolytic drug VPA

We previously showed that larvae treated with VPA [2 mM] (VPA_2_) had an altered PDR with overall lower swimming activity.^30^To assess a possible involvement of CB2, we set up parallel VPA_2_ treatments of wild-type and mutant larvae. After recording, we graphed the averaged traveled distance/larva/min (Fig. 3A, *N*=5, untreated Wt black *n*=40; untreated KO red *n*=40; Wt_VPA2_ blue *n*=80; and KO_VPA2_ orange *n*=80). All VPA_2_-treated larvae were exhibiting a strong overall decreased activity similar at most recorded time points, independently of the genotype. We found a few significant differences with mutant traveling less than wild-type animals in D/L post-transitions (Fig. 3B, successive: left panel white, *p*<0.01; cumulative: right panel white Wt_VPA2_ blue=4.82 cm/min vs. KO_VPA2_ orange=2.77 cm/min, *p*<0.0001). Also, in D periods, treated mutant traveled slightly but significantly more than treated wild-type larvae in nontransitions (Fig. 3C, successive: left panel gray, *p*<0.01 except D4; and cumulative: right panel gray Wt_VPA2_ blue=4.06 cm/min vs. KO_VPA2_ orange=5.16 cm/min, *p*<0.005). Thus, these results suggested that VPA-triggered decreased swimming activity was partially modulated by CB2.

Inner traveled distances in D periods were reduced in both treated wild-type and mutant (Fig. 3D, left panel gray, *p*<0.01) and the CO was reduced (right panel gray box CO_Wt_ black=20.26% vs. CO_Wt-VPA2_ blue=13.94%, *p*<0.001; CO_KO_ red=13.94% vs. CO_KO-VPA2_ orange=5.75%, *p*<0.0001). In L periods, the CO was also significantly reduced in treated versus nontreated mutants (white, CO_KO_ red=3.42% vs. CO_KO-VPA2_ orange=0.99%, *p*<0.0001). Thus, CO was strongly decreased in all treated animals and even more so in CB2-KO animals.

### Animals lacking CB2 respond differently to anxiogenic drug PTZ

**FIG. 4.**
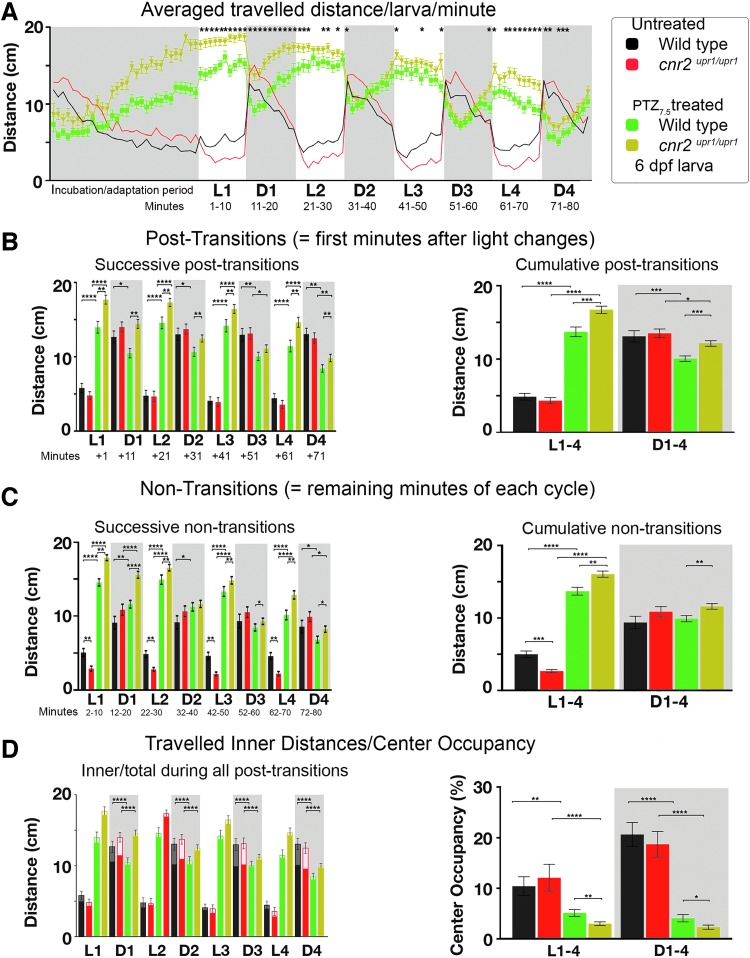
PDR in untreated and PTZ [7.5 mM] (PTZ_7.5_)-treated wild-type and *cnr2^upr1/upr1^* 6-dpf larvae. **(A)** Averaged distance traveled per minute by PTZ_7.5_-treated wild-type (green line and squares) and PTZ_7.5_-treated *cnr2^upr1/upr1^* (khaki line and triangles), and by untreated wild-type (black line) and *cnr2^upr1/upr1^* larvae (red line) represented in filigree. **(B)** Distances traveled in all successive (left panel) and cumulative (right panel) post-transitions by untreated wild-type (black bars) and *cnr2^upr1/upr1^* (red bars), and PTZ_7.5_-treated wild-type (green bars) and *cnr2^upr1/upr1^* (khaki bars) larvae. **(C)** Distances traveled during all successive (left panel) and cumulative (right) nontransitions. **(D)** Inner distances (lighter color) traveled in successive nontransitions sur-imposed on total traveled distances (left panel). Percentage of CO calculated as follows: inner/total traveled distance×100. Error bars represent SEM. Statistical significance in **(A)** compares treated wild type versus treated mutant, in all other graphs as indicated by bracket, *p<0.05, **p<0.01, ***p<0.001, and ****p<0.0001, and for clarity ns is omitted. PTZ, pentylenetetrazol

To assess a possible involvement of CB2 in modulating the previously described inverted PDR induced by PTZ_7.5_,^30,34,40^ we set up parallel PTZ_7.5_ treatments in wild-type and CB2-KO larvae. We graphed averaged traveled distance/larva/min (Fig. 4A, *N*=6, untreated Wt black *n*=56; untreated KO red *n*=56; Wt_PTZ7.5_ green *n*=96; and KO_PTZ7.5_ khaki *n*=96). All PTZ_7.5_-treated larvae displayed the expected inverted PDR: stark hyperactivity in L, which was momentarily decreased in D periods independently of genotype. However, in L periods, hyperactivity was significantly stronger in mutants in all D/L post-transitions (Fig. 4B, successive: left panel white, *p*<0.01; and cumulative: right panel white Wt_PTZ7.5_ green=13.56 cm/min vs. KO_PTZ7.5_ khaki=16.55 cm/min, *p*<0.001) and also in nontransitions (Fig. 4C, successive: left panel white, *p*<0.001; and cumulative: right panel white Wt_PTZ7.5_ green=13.56 cm/min vs. KO_PTZ7.5_ khaki=16.55 cm/min, *p*<0.001). Thus, in the absence of CB2, the PTZ-induced hyperactivity in L was enhanced. In D periods, all treated larvae strongly reduced hyperactivity immediately after a light change. However, the reduction was smaller in mutants in post-transitions (Fig. 4B, successive: left panel gray *p*<0.01 except D3; and cumulative: right panel gray Wt_PTZ7.5__=_ 9.93 cm/min vs. KO_PTZ7.5__=_ 11.98 cm/min, *p*<0.0001), and in nontransitions (Fig. 4C, successive: left panel gray *p*<0.05 except in D2; and cumulative: right panel gray Wt _PTZ7.5__=_ 9.79 cm/min vs. KO_PTZ7.5__=_ 11.49 cm/min, *p*<0.01). Thus, in the absence of CB2, the PTZ-induced hypoactivity in D was decreased.

Inner traveled distances were strongly reduced in all PTZ_7.5_-treated animals, but only in D periods (Fig. 4D, left panel, gray boxes, *p*<0.0001). The CO was strongly reduced in L (right panel white box CO_Wt_ black=10.38% vs. CO_Wt-PTZ_ green=5.04%, *p*<0.0001; and CO_KO_ red=12.06% vs. CO_KO-PTZ_ khaki=2.91%, *p*<0.0001) and in D periods (gray box CO_Wt_ black=20.68% vs. CO_Wt-PTZ_ green=4.00%, *p*<0.0001; and CO_KO_ red=18.72% vs. CO_KO-PTZ_ khaki=2.21%, *p*<0.0001). Remarkably, the CO reduction was even more pronounced in CB2-KO animals. Taken together, PTZ-triggered inverted PDR and decreased CO were significantly altered in the absence of CB2, suggesting that CB2 was a modulator of the PTZ anxiogenic effects.

### A subset of CB2 ligand activity alters the PDR differently in wild-type and CB2-KO larvae

**FIG. 5.**
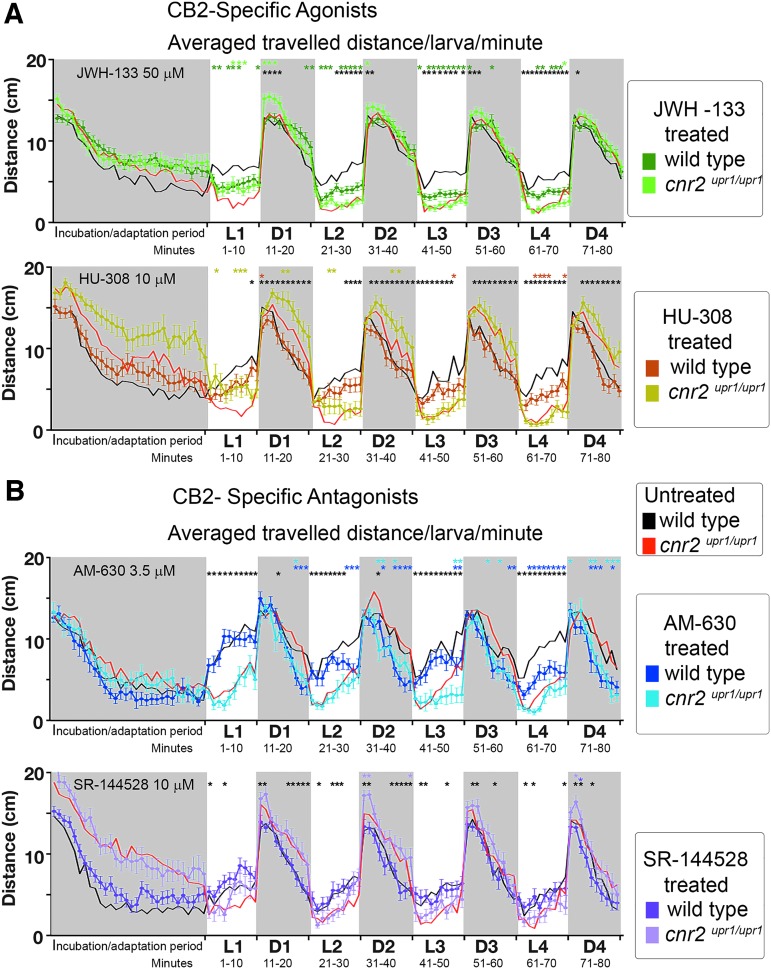
PDR in untreated and CB2 agonist (JWH-133 and HU-308)- and CB2 antagonist (AM-630 and SR-144528)-treated wild-type and *cnr2^upr1/upr1^* 6-dpf larvae. **(A)** Averaged distance traveled per minute by JWH-133-treated (top panel, 50 μM) wild type (dark green line and squares) and *cnr2^upr1/upr1^* (light green line and squares); and HU-308-treated (bottom, 10 μM) wild type (brown line and squares) and *cnr2^upr1/upr1^* (khaki line and squares). **(B)** Averaged distance traveled per minute by AM-630-treated (top panel, 3.5 μM) wild type (dark blue line and squares) and *cnr2^upr1/upr1^* (light blue line and squares); and SR-144528 (bottom, 10 μM) wild type (purple line and squares) and *cnr2^upr1/upr1^* (pink line and squares). Control-untreated wild-type (black line) and *cnr2^upr1/upr1^* larvae (red line) are represented in filigree in each graph. Error bars represent the SEM. Statistical significance compares (1) treated wild type versus treated mutant=black* in all graphs, (2) untreated mutant versus treated mutant=light green* in **(A)**-top panel, khaki* in **(A)**-bottom panel, light blue* in **(B)**-top panel, and pink* in **(B)**-bottom panel, (3) untreated wild type versus treated wild type=green* in **(A)**-top panel, brown* in **(A)**-bottom panel, dark blue* in **(B)**-top panel, and purple* in **(B)**-bottom panel. *p<0.05, **p<0.01, ***p<0.001, and ****p<0.0001, and for clarity ns is omitted.

To assess if the PDR could detect CB2 ligand activity, we treated wild-type and mutant larvae with agonists (JWH-133 and HU-380) and antagonists (AM-630 and SR-144528). All ligands were pretested for overexposure-induced side effects (as detailed in the Materials and Methods section), and we analyzed further the PDR after treatment with the highest well-tolerated concentration (Fig. 5).

With JWH-133 (top panel in Fig. 5A, Wt-JWH-133_50_ dark green *n*=24, and KO-JWH-133_50_ light green *n*=24), when we compared untreated versus treated wild type (black vs. dark green), we found that treated animals traveled significantly less in L (dark green* middle row, 30/40 in L and 4/40 in D) possibly as a result of CB2 binding and activation. Furthermore, when comparing untreated versus treated mutants (red vs. light green), we found few significant differences (light green* top row, 4/40 in L and 4/40 in D), meaning there was little activity detected in the absence of CB2, suggesting a good ligand specificity and low off-target effects. Thus, we concluded that JWH-133_50_ altered PDR, possibly in a CB2-specific manner.

With HU-308 (bottom panel in Fig. 5A, Wt-HU-308_10_ brown *n*=24, and KO-HU-308_10_ khaki *n*=24), when we compared untreated versus treated wild type (black vs. brown), we found very few significant differences (brown*middle row, 6/40 in light and 1/40 in dark) and likewise when comparing untreated versus treated-CB2-KO larvae (red vs. khaki and khaki* top row, 6/40 in L and 4/40 in D) pointing to a weak *in vivo* effect at well-tolerated concentrations.

With AM-630 (top panel in Fig. 5B, Wt-AM-630_3.5_ dark blue *n*=24, and KO-AM-630_3.5_ light blue *n*=24), when we compared untreated versus treated wild type (black vs. dark blue), we found that treated animals traveled less during nontransitions (dark blue* middle row 13/40 in L and 14/40 in D), possibly as a result of CB2 binding and activation. When comparing untreated versus treated mutant larvae (red vs. light blue), we found a few significant differences mostly in D periods (light blue*, top row 2/40 in L and 12/40 in D), suggesting that the detectable AM-630_3.5_-induced *in vivo* effect might be CB2 specific in L, but be off-target effects in D periods.

With SR-144528 (bottom panel in Fig. 5B, Wt-SR-144528_10_ purple *n*=24 and KO-SR-144528_10_ pink *n*=24), when we compared untreated versus treated wild type (black vs. purple), we found no significant differences (purple* middle row 0/40 in L and 1/40 in D) and likewise with untreated versus treated mutant larvae (red vs. pink, and pink* top row 2/40 in L and 12/40 in D) pointing to a weak *in vivo* effect at well-tolerated concentrations. Taken together, we elicited detectable *in vivo* effects with two of four tested CB2-ligands, which altered the PDR significantly at a subset of time points in wild type but not in mutants, arguing for CB2 specificity of the observed effects.

## Discussion and Conclusions

To assess CB2 involvement in complex behaviors during vertebrate development, we generated with CRISPR-Cas9 technology CB2-KO animals and tested homozygote (*cnr2^upr1/upr1^*) larvae in a PDR swimming behavior assay. We showed that mutant animals were swimming significantly less in light, more in the dark, and avoiding open spaces more than wild type. Thus, we provide evidence for CB2 involvement in complex larval behaviors.

Hyperactivity and hypoactivity associated with, but not limited to, light changes are well-accepted measures of anxiety-like behaviors in rodents and have been also explored in adult fish^41–43^ although not yet extensively in larvae.^44,45^ We tested 6-dpf larvae because at this developmental stage, animals swim upright and exhibit complex behaviors comparable with adults. Using larvae presents major experimental advantages such as enabling upscalability. The small size (∼2 mm) and relative permeability of young larvae simplify chemical treatments that can be simply added to the water and will penetrate the animal by simple diffusion. Weekly spawning (∼100 eggs/couple/week) can provide ample number of animals for parallel testing of various concentrations of compounds. Center avoidance is another classical measure of anxiety-like behavior.^46,47^ As described previously, wild-type larvae swim mostly near the walls but travel more in the center during L/D post-transitions.^29^ So, we measured inner traveled distances in post-transitions. However, variation of inner distances might simply reflect variation of the total activity, so we expressed relative distances traveled as a ratio: inner traveled distance/total traveled distance×100 to obtain the percentage of CO, providing an inverse reading of center avoidance. Therefore, we established anxiety-like *in vivo* parameters for fish that open new experimental avenues.

Next, we showed that when treating larvae with the anxiolytic drug VPA, the PDR was strongly reduced similarly in wild-type and mutant larvae, but with stronger hypoactivity in D/L post-transitions and more activity in dark nontransitions in the latter. VPA (or valproate) is a broad-spectrum anxiolytic drug^31^ that increases gamma-aminobutyric acid (GABA) turnover, inhibits glutamate/N-methyl-d-aspartate (NMDA) receptors, and blocks voltage-dependent sodium channels.^48–50^ The complex mode of action in a whole organism is yet to be clarified, and our results argue for only a marginal modulation by CB2. Surprisingly, the CO was strongly reduced in darkness indicating that all treated animals were avoiding open space. Those results were suggesting that VPA had an anxiogenic effect in fish larvae, which was amplified in the absence of CB2. Alternatively, this might reflect sedation, a commonly described side effect in VPA.^49^

PTZ is commonly used in animal models to induce anxiety and seizure-like activity that is principally mediated via GABA_A_ inhibition.^35,51^ We and others have previously shown that with a fixed concentration of PTZ [7.5 mM] (PTZ_7.5_), a strong inverted PDR could be induced in wild-type larvae, namely hyperactivity in light periods and hypoactivity in dark periods.^30,34,40^ Treated CB2-KO larvae had consistent heightened inverted PDR in light periods. Involvement of CB2 in the PTZ-elicited GABA_A_ inhibition was previously shown in rodents^52^ and offers a potential explanation. However, a greater sensitivity to treatments of mutant larvae, as well as possible additive effects occurring in parallel signaling pathways, cannot be excluded at this point. Testing of different doses of VPA and PTZ as well as cotreatments will help elucidate CB2 involvement.

We also measured the effect on the PDR with four known CB2 ligands and showed that we elicited with two of them, PDR alterations that were possibly CB2 mediated. Our data are proof of principle that such an approach could be further developed into an effective means to screen ligand-binding efficacy, specificity, as well as drug safety in a whole organism. However, a few major drawbacks must be addressed before exploiting this approach on a large scale. First, possibly because of the mode of administration of the ligands (directly into the water), we had to use very high concentrations of ligands to elicit a detectable response. This significantly narrowed the testable range of concentrations before reaching toxic levels. Alternative means of drug administration should be explored such as food additives. Second, we found an internal variation of the PDR across experiments in untreated wild-type and mutant larvae alike, rendering phenotypic differences in dark periods less consistent across experiments with different treatments especially when using smaller sample size (*n*<40). However, we found that the robustness of the phenotype could be easily strengthened by augmenting the number of tested animals, and by always using nontreated wild-type and mutant animals from the same clutches in parallel runs to provide solid internal controls.

In summary, we present an innovative upscalable approach that can be coupled to automated readout for significant differences and applied to drug discovery pipeline to test new CB2 ligand lead compounds. Likewise, mutant lines for CB1, opioid receptors, or GABA subunits could be established and double or even triple-KO lines used as screening tools. Finally, the indispensable preclinical safety and efficacy testing needed for bringing new drugs to the market could be performed in zebrafish larvae, offering a cost-effective, fast, and easy alternative or complement to the more classical preclinical models.

## Abbreviations Used

aa: amino acids
Ab: antibody
CO: center occupancy
dpf: day postfertilization
eCB: endocannabinoid
GABA: gamma-aminobutyric acid
INDEL: insertion/deletion
NMDA: N-methyl-d-aspartate
PCR: polymerase chain reaction
PDR: photo-dependent responses
PTZ: pentylenetetrazol
SEM: standard error of the mean
VPA: valproic acid
